# Power-Assisted Liposuction (P.A.L.) Fat Harvesting for Lipofilling: The Trap Device

**Published:** 2015-07

**Authors:** Denis Codazzi, Stefano Bruschi, Enrico Robotti, Maria Alessandra Bocchiotti

**Affiliations:** 1 Department of Plastic Surgery, University of Turin, San Giovanni Battista Hospital, Turin, Italy;; 2 Department of Plastic Surgery, Papa Giovanni XXIII Hospital, Bergamo, Italy

**Keywords:** Power Assisted Liposuction, Fat grafting, Trap device, Lipofilling, Italy

**DEAR EDITOR**

Coleman classically described four phases in fat grafting including harvesting, refinement, transfer and placement. The harvesting phase can be simplified by using the “trap device” instead of the conventional 10-cc Luer-Lock syringes. The “trap device” for harvesting fat by Power Assisted Liposuction and Medinorm tank is a convenient, wholly sterile, time saving method to provide fat for lipostructure in various part of the body.^1^ The harvesting phase can be simplified by using Power-Assisted Liposuction (P.A.L.) instead of the conventional 10-cc Luer-Lock syringes.^2^ This study evaluated the trap device of P.A.L. in fat harvesting for lipofilling. 

MicroAire P.A.L.^TM^ (MicroAire Surgical Instruments LLC, 1641 Edlich Drive Charlottesville, VA 22911) is an electric device for liposuction, composed of a motorized handle (mod. 600-E) connected to an aspiration cannula of varying size and types, a standard plastic tubing for fat aspiration, and an electric control console (mod. 1020). The P.A.L. produces oscillating reciprocal “to-and-fro” movements of the cannula tip^2^ with a 2,4 millimetres stroke and a 0 to 4500/min vibration range. 

We thought the same system would find ready application to fat harvesting if an efficient sterile fat trap could be fashioned. Thus, instead of directly connecting the plastic suction tube to a common aspirator, we deviced a trap composed of the reservoir of an “High Vacuum Wound Drainage System” of 600 cc (Medinorm Medizintechnik GmbH, Gewerbepark 7–9, D-66583 Spiesen-Elversberg, Germany) deprived of its two rubber caps. The P.A.L. tube was connected to the longest of the two beaks of the 600 cc reservoir by using the forefinger of a surgical glove as a gasket (both tube and beak were stiff and they otherwise would not fit one another); the other beak was directly connected with the tube of the customary aspirator. To avoid leaks and loss of suction, two small Opsites^ TM^ (Smith and Nephew, Inc 1450 E Brooks Rd Memphis, TN 38116) were used as an “insulating tape” around the two connections. The P.A.L. harvesting cannulas that we employed were a single-hole MicroAire of 3 mm in diameter (PAL-R300LS single-port) and a triple-hole MicroAire of 4 mm (PAL-R402LS Triport II) (Figure 1).

**Fig. 1:**
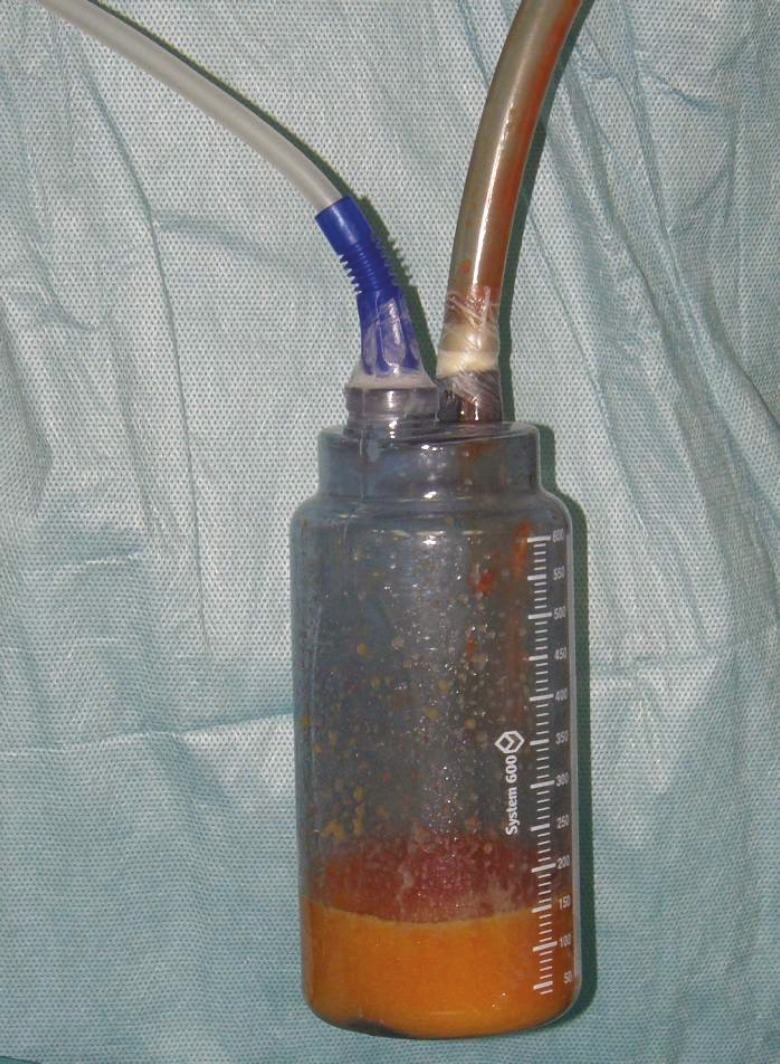
The trap device: the wider bore tube on right side brings fat from the P.A.L. handle to the reservoir, while the other smaller bore one on the left connected the tank to a common aspirator

In a 38 years old patient (1.75 cm tall, weighing 57 kg, with a body mass index of 18.6) undergoing lipostructure for a severe pectus excavatum, we easily harvested high quality fat from both medial knees and the abdomen, after infiltration with 500 ml of a modified Klein formula (a dilution solution of 1% lidocaine, 50 ml), 1% adrenaline (1 ml) and 8,4% bicarbonate (2.5 ml) in 1000 ml of physiologic saline), 600 ml (13 ml per minute). After centrifugation according to Coleman, the pure yellow fat at our disposal was 400 ml that was two thirds of the initial harvested volume. The harvesting and refinement procedure took 40 minutes. An electric microair device was employed, with a relatively low vibration range of 0-1,350, as adjusted on the control console and on the handle.

The P.A.L. device is gaining popularity in liposuction procedures. The oscillating movement of the cannula easily mimics, in a strainless and more controlled fashion, the work of a surgeon during traditional suction-assisted lipoplasty. The vibration allows easy penetration of even fibrous fat, while generating no thermal energy, thus no risk of cutaneous burns as compared with ultrasound liposuction.^2^

The rate of fat extraction is fast, while surgeon fatigue and intraoperative time are decreased, possibly also accounting in reduced intraoperative pain, postoperative edema and ecchymoses. Traditional difficulty in contouring certain areas (e.g. the periumbilical region) can be overcome by simply stabilizing the position of the cannula and allowing the reciprocating tip to remove fat.^3^ Drawbacks are an obvious learning curve (after performing eight or more cases of P.A.L., surgeons harvest 45% more fat per minute in comparison with traditional liposuction) and, of course, the price.^4^

Lipostructure by harvesting fat as described by Coleman^1^^,^^5^^,^^6^ is also rapidly gaining popularity worldwide, both as a stand-alone procedure and as an adjunct to other interventions, with wide both reconstructive and cosmetic indications. For instance, in our institution, we now add some form of lipofilling to practically any surgical step when reconstructiung a breast.^1^^,^^5^^,^^6^ It is to be noted that, especially for lipostructure in breast reconstruction, significant amounts of fat are often needed. 

The P.A.L. system can be used for harvesting fat by fashioning a trap as described above.^5^ Medinorm’s tank and the tubing system guarantee the sterility that would be lost when collecting the fat directly from the aspirator tank. Also, the whole system is latex allergen free. It also seems to us that the harvested fat is less bloody than what we usually see after manual liposuction that indeed the fat collected in the trap is already of almost pure yellow color.

The low cost of the Medinorm drain of 600 ml reservoir (9 Euros) is an added bonus, with the possibility of replacing the reservoir with a new one when greater amounts of fat are needed, while other members of the team proceeded with refining the contents of the first bottle. Fat transfer from the trap to the 10 ml syringes that will then be inserted into the centrifuge takes a few seconds: after both tubes are removed from the bottle, the fat can simply be poured to fill each syringe. Additionally, this method preserves fat from ambient air exposure.

Finally, it could be argued that this system is more traumatic to fat cells than the careful, low-pressure extraction via a Coleman harvesting cannula and a 10 ml syringe. We however believe that it is not so that the control rotating knob on the electric console directly administered (as shown by the number of blue light LEDs) the cannula top vibration range, while the hand piece allows further fine regulation of such range within the presaid parameters (for instance, three visible blue light LEDs administer a maximum cannula vibration range of 1,350 vibs/min; the hand piece can be then used, in proportion to digital pressure, to finely tune such range from 0 to indeed 1,350) (Figure 2).

**Fig. 2:**
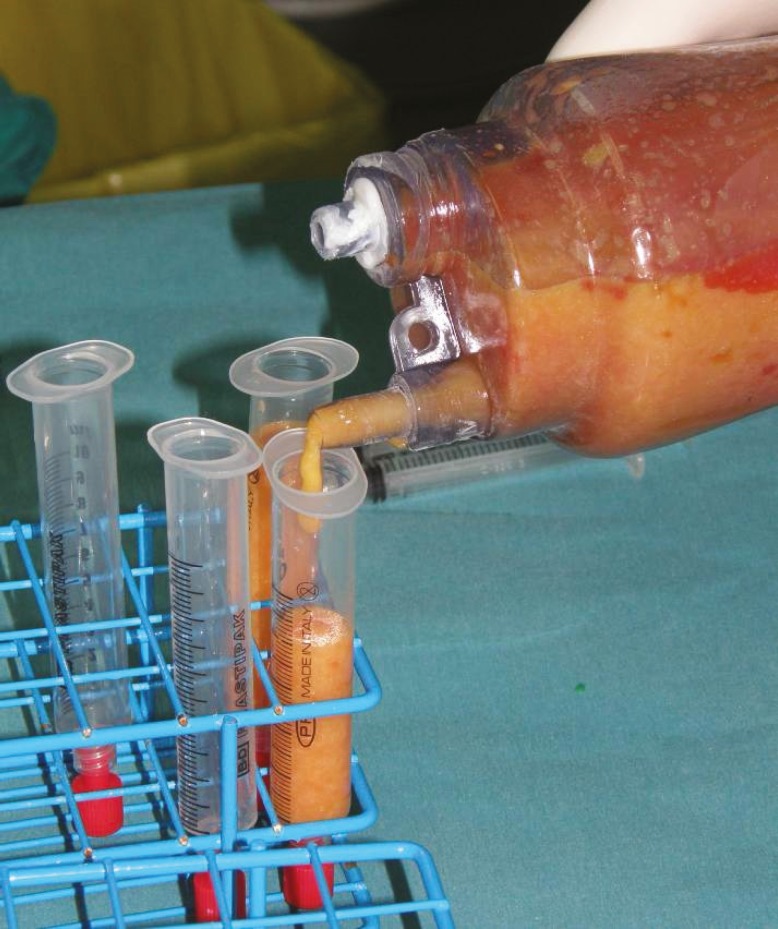
Fat transfer from tank to syringes which will be centrifuged.

We can conclude that the “trap device” for harvesting fat in P.A.L. liposuction is a convenient, wholly sterile, time saving method to provide fat for lipostructure in various part of the body. Time is saved and donor site morbidity minimized.

## CONFLICT OF INTEREST

The authors declare no conflict of interest.

## Note

Please cite this paper as:

Codazzi D, Bruschi S, Robotti E, Bocchiotti MA. Power-Assisted Liposuction (P.A.L.) Fat Harvesting for Lipofilling: The Trap Device. World J Plast Surg 2015;4(2):177-179.
